# Identification of the IDA peptide family in tomato and function of *SlIDA8* in salt stress

**DOI:** 10.1186/s12870-026-08179-5

**Published:** 2026-01-20

**Authors:** Xintong Song, Qiaomei Ma, Jiamiao Wu, Yuxiang Hu, Zhenqing Zhao, Zhanming Tan

**Affiliations:** 1 College of Horticulture and Forestry, Tarim University, Alar, 843300 China; 2 Xinjiang Production & Construction Corps Key Laboratory of Protected Agriculture, Alar, China; 3 Vegetable Research Institute of Zhejiang Academy of Agricultural Sciences, Zhejiang, 325005 China

**Keywords:** Tomato, Salt stress, IDA polypeptide family, Gene family analysis, Gene function validation

## Abstract

**Background:**

Research on the flocculation-deficient abscission peptide IDA has mostly concentrated on how it controls the growth and development of plants. However, research on the role of defectin in tomato (*Solanum lycopersicum*) under salt stress remains limited.

**Results:**

Through analysis of the tomato genome, we identified a total of 11 SlIDA peptides and classified them into three categories based on their phylogenetic relationships. These genes exhibit chromosomal duplication events. Further structural analysis revealed that members of this gene family possess a high degree of evolutionary conservation. Expression analysis revealed that all 11 *SlIDA* genes are expressed in various tomato tissues, with their expression levels exhibiting significant changes in response to salt stress. Among them, *SlIDA8* demonstrated the most pronounced upregulation, suggesting a potential key role in salt stress response. Functional validation demonstrated that the SlIDA8 protein localizes to both the cytoplasm and nucleus. Comparing the performance of *SlIDA8*-silenced plants with wild-type plants under salt stress revealed significantly reduced salt tolerance in the silenced plants. This result directly confirms that *SlIDA8* is an important positive regulator in the tomato response to salt stress.

**Conclusions:**

Results indicate that *SlIDA8*-silenced plants exhibit a more pronounced stress phenotype, characterized by accelerated chlorophyll degradation, increased membrane damage, reduced activity of reactive oxygen species scavenging enzymes, and elevated abscisic acid (ABA) content. This suggests that *SlIDA8* silencing enhances tomato sensitivity to salt stress. These findings preliminarily elucidate the role of *SlIDA8* as a positive regulator of salt stress in tomatoes providing a research basis for further investigation into IDA function.

**Supplementary Information:**

The online version contains supplementary material available at 10.1186/s12870-026-08179-5.

## Background

Tomato (*Solanum lycopersicum* L.), an annual herbaceous plant of the genus Solanum in the family Solanaceae, is native to South America and contains various nutrients [1, 2]. China is one of the world's largest producers and consumers of tomatoes, but it is often affected by several biotic and abiotic stresses, including salt stress, which has considerable impacts, seriously affecting the yield of tomato fruit and seriously impairing its quality [3]. Salt stress seriously affects plant growth and development, and its main effects include osmotic stress, ionic injury, nutrient deficiency, and the hindrance of photosynthesis and metabolic processes [4]. Therefore, when plants are subjected to salt stress, they defend themselves through various strategies, such as osmotic regulation, ion homeostasis, and scavenging of reactive oxygen species. Phytohormones also play significant roles in plant responses to salt stress [5–7]. When plants are subjected to biotic and abiotic stresses, phytohormones, which are important small signaling molecules in plants, enhance plant tolerance by promoting plant cell division, directing specific differentiation programs, ion homeostasis, and antioxidant enzyme activities [6, 7]. For example, plant hormones, such as abscisic acid (ABA), jasmonic acid (JA), oleuropein lactone, and ethylene (ETH), enhance plant salt tolerance and reduce the negative effects of salt stress by regulating stomatal closure, sodium and potassium ion homeostasis, and antioxidant enzyme activity [8, 9].

Recently, plant polypeptides have been recognized as a new type of plant hormone. Polypeptides are formed by two or more α-amino acids linked together by peptide bonds; plant polypeptides are composed of 5–60 amino acid residues that affect plant growth and development by binding to receptors and are active at very low concentrations [10]. Since the discovery of the first peptide and its phylogeny in tomatoes, many studies have shown that peptides play important roles as signaling molecules in plant growth, development, and defense responses [11]. Peptide signaling molecules have become important mediators in the signaling processes of plant cellular components [12]. Moreover, with further research, it has been found that plant peptides also play a pivotal role in plant resistance pathways to adversity stress. For example, SlCEPs (Solanum lycopersicum C-terminally encoded peptides) significantly enhance tomato drought tolerance [13], and current research indicates CEPs play a role in potato nitrogen deficiency [14]; *NtCLE3* not only improves tobacco survival rates and antioxidant capacity under drought stress but also enhances drought resistance in *Solanaceae* crops such as sweet peppers and eggplants [15].

INFLORESCENCE DEFICIENT IN ABSCISSION (IDA) is a secreted peptide of 77 amino acid residues that binds to receptor-like kinases and plays a role in floral organ abscission and lateral root sprouting [16, 17]. In *Arabidopsis*, nine members of the IDA peptide family have been identified, including IDA and its analogous peptides IDA LIKE (IDLs) 1–8, of which six members, IDA and IDL1-5, are highly conserved at the C-terminal end [18]. The IDA and IDL1-5 polypeptides have a secretion signal at the N-terminus and a conserved PIP motif at the C-terminus. N-terminal end of the PIP motif and a conserved variable number of additional amino acid sequences. The PIP motif is the main functional structural domain of the IDA and its IDL polypeptides [16]. The IDA polypeptide functions mainly through the LRR-RLK, HAESA (HAE), and HAESA-L1KE2 (HSL2) receptors. Current studies of the IDA polypeptide family have mostly focused on the regulation of plant growth and development, such as flower abscission and lateral root growth. The functions of IDA/IDLs have been extended to various biological processes, including plant growth, development, and abiotic responses. IDA polypeptides in *Arabidopsis* respond to abiotic stresses, such as salt stress and cold stress [19, 20]. However, the functional analysis of IDA polypeptides in tomatoes in response to salt stress has not been documented as of yet.

Therefore, this study aimed to identify the IDA peptide family in tomatoes and analyze its role in salt resistance. In this study, we identified and analyzed the tomato IDA polypeptide family using bioinformatics techniques, including phylogenetic construction, chromosomal distribution, and gene structure analysis. The expression profiles of tomato IDA polypeptide family genes under salt stress were analyzed using qRT-PCR, and *SlIDA8*, which is more sensitive to salt stress, was screened. In addition, *SlIDA8* was silenced by virus-induced gene silencing (VIGS). Therefore, this study provides a theoretical foundation for further exploration of the functional mechanisms of the SlIDA family, while also offering new potential targets and strategies for future improvement of tomato tolerance to salt stress.

## Materials and methods

### Plant materials and salt stress treatments

#### Plant materials

All plant materials were grown in an artificial climate chamber at the Zhejiang Academy of Agricultural Sciences (Hangzhou, Zhejiang Province, China) under day/night temperatures of 25/21 °C, a photoperiod of 14/10 h, and light intensity of 500 µmol m^−2^ s^−1^. Five-week-old tomato plants were used in the subsequent experiments.

Control experimental material: The wild-type tomato variety (*Solanum lycopersicum cv. Condine Red*) was used as control plant material, which was a laboratory-retained seed stock. Seeds were germinated in a shaker at 28 °C until dewy, sown into cavity trays, and transplanted to small pots (substrate of grass carbon and vermiculite in a ratio of 2:1 by volume) for 14 d, about 2 weeks, later for the appropriate treatments.

Experimental group materials: By creating particular primers for particular SlIDA8 segments, VIGS gene silencing plant constructs were created and amplified by PCR (Supplemental Table S1). By incorporating the *SlIDA8* fragment into the tobacco crisped-virus RNA2 (TRV2) vector and exposing it to *Agrobacterium tumefaciens* GV3101, the recombinant vector TRV2: *SlIDA8* was created. TRV1-containing Agrobacterium tumefaciens was combined in a 1:1 volume ratio with TRV2: *SlIDA8*, pTRV2: *00* (negative control), or pTRV2-*SlPDS* (positive control) vectors. Agrobacterium suspension (OD600 = 1.2–1.6) was used to infiltrate two-leaf stage tomato plants. When the leaves of pTRV2: *SlPDS* plants showed a photobleaching phenotype, the expression of *SlIDA8* in TRV2: *SlIDA8* plants was detected using qRT-PCR, and the silencing efficiency was calculated.

#### Stress treatments

Roots, stems, leaves, flowers, and green-ripe stage fruits of WT plants were collected and snap-frozen in liquid nitrogen, with sampling replicates of *n* = 4. The samples were used to analyse the tissue expression specificity of IDA polypeptide family genes in tomatoes.

Salt tolerance analysis by hydroponics: Tomato seeds were shaken at 9:00 a.m., sterilized at 5:00 p.m., shaken until the seeds were dewy, and the dewy seeds were cultured in 1/2 MS medium for 10 d, after which they were transplanted to hydroponic 1/2 concentration of Hogan's nutrient solution; 12 d after transplanting, they were subjected to salt treatment, which was performed by treating them with 1/4 concentration of Hogan's nutrient solution with 150 mM NaCl. After 6 h of salt treatment, 3rd leaf position, leaf, and root tip samples were collected and snap-frozen in liquid nitrogen with *n* = 4 replicates to conduct genetic screening for response to salt stress.

Salt tolerance analysis was performed using the rooting method: tomato seeds were soaked in warm broth (55 °C, 30 min), germinated in a shaker at 28 °C until dewy, sown into hole trays, and transplanted into small pots (substrate of grass carbon and vermiculite in a volume ratio of 2:1) approximately 2 weeks later. Four-week-old tomatoes were rooted in 200 mM NaCl (50 mL/pc) and sampled 48 h after the treatment. Leaves at 3 and 4-leaf positions were collected, veins were removed, and each sample was weighed at 0.2 g. Four replicates were used in *n* = 4. Samples were used for RNA extraction and antioxidant enzyme activity assays. Afterwards, the corresponding treatment solution was increased every 3 d until the salt damage phenotype appeared, and chlorophyll fluorescence and relative conductivity assays were performed.

### Genomic identification of the *SlIDA* gene in tomato

We obtained the IDA protein sequence in *Arabidopsis* from the TAIR database (https://www.arabidopsis.org/) and used it as a target sequence to identify the *SlIDA* gene in the tomato genome. We searched the tomato genome database (https://solgenomics.ösearch/locus) and Ensembl Genome (https://plants.ensembl.org/index.html) to identify *SlIDA*. In addition, their physicochemical characteristics (molecular weight and isoelectric point) were calculated using ExPASy (https://web.expasy.org/compute_pi/).

### Chromosome localisation, conserved motif analysis, and gene structure

Based on the chromosomal localization information of the *SlIDA* gene provided by the SGN website, the chromosomal localization of the *SlIDA* gene was schematically drawn using MG2C (http://mg2c.iask.in/mg2c_v2.1/). The gene structure of *SlIDA* was mapped using the Gene Structure Display Server (http://gsds.cbi.pku.edu.cn/), Multiple Covariance Scanning Toolkit (MCScanX), and the gene duplication events of *SlIDA* were checked using the default parameters [21]. Conserved motif analysis of IDA proteins was performed using the MEME tool (https://meme-suite.org/meme/). The following parameters were used in this study: the maximum number of motifs was 10, and the width of the motifs ranged from 6 to 200 residues.

### Phylogenetic analysis

Sequence comparisons of IDA proteins from tomato, *Arabidopsis*, *Nicotiana tabacum*, and *Solanum tuberosum* were performed using ClustalW and the integrated tool MEGA11. The phylogenetic trees were constructed by the neighbour-joining (NJ) algorithm in the software, in which the bootstrap repeat value was set to 1000. Phylogenetic trees were constructed using a mapping website (https://itol.embl.de/) [22].

### Tomato total RNA extraction and qRT-PCR analysis

Frozen plant tissues were rapidly shocked in liquid nitrogen, and the frozen samples were ground using a pre-cooled grinder. Total RNA was extracted from tomato tissues using a total RNA extraction kit (DP230410, TIANGEN BIOTECH, Beijing, China) according to the manufacturer’s instructions and was reverse-transcribed into cDNA after completion of the extraction for subsequent detection of relative gene expression. Fluorescent quantitative primers were designed on the Primer 3 website, and detailed data are shown in Supplemental Table S1. qRT-RCR assays were performed using the SYBR qPCR SuperMix enzyme, and the relative expression was calculated using the 2^−ΔΔCt^ method. The ACTIN gene was used as a standardised internal reference, and four biological replicates were set up for each sample. The qRT-PCR reaction system was as follows: 0.4 μL of forward primer, 0.4 μL of reverse primer, 1 μL of diluted cDNA samples, and 10 μL of 2 × ChamQ Universal SYBR qPCR Master Mix, and finally, sterile ultrapure water was added to replenish up to 20 μL. The conditions were: pre-denaturation at 94 °C for 30 s, denaturation at 94 °C for 5 s, and annealing and extension at 60 °C for 30 s, with a total of 32 cycles.

### Protein subcellular localisation

*SlIDA8*'s subcellular location was examined by amplifying its coding sequence (without a stop codon) using PCR and cloning it into the GFP-containing expression vector PAC402 by homologous recombination, which was regulated by the CaMV35S promoter. The histone 2B-mCherry vector was used as the nuclear marker. The constructed plasmid was transfected into *A. tumefaciens* strain GV3101, and the fusion construct was transformed into tobacco leaves. After 48 h of transfection, GFP fluorescence was detected using a laser confocal microscope (Carl Zeiss, Thornwood, NY, USA) [23]. The subcellular localisation primers are listed in the Supplemental Table S1.

### Determination of antioxidant enzyme activity

After 48 h of salt stress, 0.2 g of leaves were collected and homogenised with 2 mL of ice-cold 50 mM phosphate buffer (pH 7.8, 0.2 mM EDTA, 2% (w/v) polyvinylpyrrolidone, and 2 mM L-ascorbic acid). The homogenate was centrifuged at 4 °C, 12,000 g for 20 min, and the resulting supernatant was used for assaying the enzyme activity.

MDA is one of the end products of the lipid peroxidation reaction, which is often used to assess the level of oxidative stress in organisms and has a maximum absorption peak at 532 nm. POD can catalyse the oxidation of phenolic and amine compounds by hydrogen peroxide, which has the dual effect of eliminating the toxicity of hydrogen peroxide and phenolic and amine compounds and has the characteristic of a specific absorption peak at 470 nm for the determination of its content in plant tissues. CAT is the most important H_2_O_2_ scavenging enzyme, and H_2_O_2_ has a characteristic absorption peak at 240 nm; therefore, the absorbance of the reaction solution at 240 nm decreases with reaction time, and the CAT activity can be calculated according to the rate of change of absorbance. SOD is a metalloenzyme that is widely present in living organisms and is an important oxygen radical scavenger, which produces superoxide anion (O_2_^−^) through the reaction of xanthine and xanthine oxidase; the reduction product of O_2_^−^, methyl filth, has a characteristic absorption peak at 560 nm. In accordance with these experimental principles, the MDA content and the activities of three enzymes, CAT, SOD and POD, were determined using an assay kit (Solarbio, China), with the methodology described below [24].

The production of hydrogen peroxide (H_2_O_2_) and superoxide anion (O_2_^−^) in leaves was then detected using 3, 3′-diaminobenzidine (DAB) and nitro tetrazolium blue chloride (NBT) staining methods, respectively, following the same methods as Gong [25].

### Determination of relative electrolyte leakage rate and chlorophyll fluorescence

Overall, 0.3 g of tomato leaves was weighed, the leaves were washed with ddH_2_O, the main veins of the leaves were removed, the leaves were cut into 1 cm^2^ pieces, placed in 50 mL test tubes, and 20 mL of ddH_2_O was added. After shaking at 200 rpm for 2 h at room temperature, conductivity measurements were performed and recorded as EL1. The samples were subjected to a water bath heating at 95 °C for 15 min and then cooled down to room temperature. The conductivity was measured again and recorded as EL2, and the relative electrolyte permeability was calculated according to the following equation. Four replicates were performed per treatment group was 4 [26].

$$\text{Ethylene }\left(\%\right)=\mathrm{EL}1/\mathrm{EL}2\times 100$$

The maximum photochemical efficiency (Fv/Fm) of PSII in leaves was determined as described [27, 28].

#### Plant hormone assay

For ABA measurement, collect 0.1 g of leaf tissue 6 h after salt stress application. Plant hormone extraction and analysis were performed according to the previously established method [28, 29].

### Statistical analysis

For multiple comparisons satisfying normality and homogeneity of variance assumptions, one-way analysis of variance (ANOVA) was performed. If ANOVA results indicated significant differences (*p* < 0.05), Tukey's Honestly Significant Difference (HSD) post hoc test was further applied for pairwise comparisons between groups. For data involving only two-group comparisons, Student's t-test was used when the same prerequisites were met. All statistical analyses were performed using SPSS software, with a significance level set at *p* < 0.05.

## Results

### SlIDA gene identification and chromosomal localization

To identify the IDA family members in tomatoes, we used the latest versions of the tomato reference genomes SL4.0 to search for *SlIDA* genes [30]. First, we performed sequence searches using the *Arabidopsis IDA* genes. Eleven *IDA* genes were identified in tomatoes, including five untagged genes: *SlIDA2*, *SlIDA3*, *SlIDA6*, *SlIDA10*, and *SlIDA11*.

**Fig. 1:**
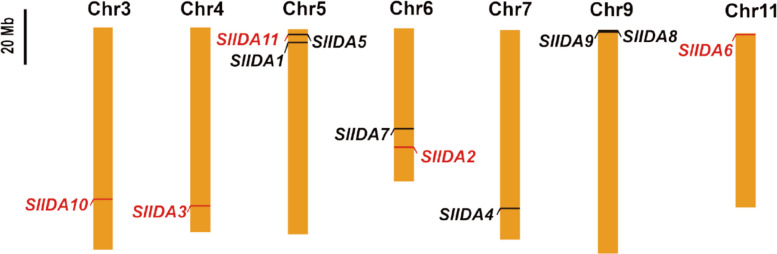
Distribution of *SlIDA* genes on tomato chromosomes. Chromosome numbers are labeled at the top of each bar graph. Red gene names indicate unmarked genes

We localized the *SlIDA* genes on the tomato (Table 1, Fig. 1) chromosomes; the five unlabeled genes are marked in red font, and the identified *SlIDA* genes were numbered sequentially. *SlIDA* genes exhibit varying degrees of expression across all seven chromosomes in tomato. These 11 genes show uneven distribution across chromosomes 3, 4, 5, 6, 7, 9, and 11. Notably, we observed multiple *SlIDA* genes-such as *SlIDA1*, *SlIDA5*, and *SlIDA11*-clustered closely together on chromosomes, forming gene clusters with highly similar sequences. This characteristic genomic structure strongly suggests that tandem duplication is the key mechanism driving the expansion and evolution of the tomato *SlIDA* gene family. During this process, the ancestral gene undergoes in situ duplication through events such as unequal crossing over. The resulting copies are tightly linked in physical position and may acquire functional diversity or specificity through subsequent sequence differentiation [31]. We further analyzed *SlIDA* for chromosome localization, amino acid length, molecular weight, and theoretical isoelectric point (Table 2). The results revealed that these proteins ranged in length from 71 to 101 amino acid residues, with isoelectric points (pI) spanning from 5.698 to 11.213 and molecular weights ranging from 7,811.218 to 12,704.135 Da.

**Table 1:** Chromosome locations and peptide sequences of SlIDAs uncovered in this study

Name	Predicted IDA	Chromosome	Start	End
SlIDA1	PIPPSAPSKRHN	chr5	4200188	4200436
SlIDA2	PIPPSAPSKRHN	chr6	38623397	38623450
SlIDA3	PIPPSSPSKRHN	chr4	57992910	57993146
SlIDA4	PIPPSAPSKRCN	chr7	58068280	58068558
SlIDA5	LIPPSGPSRRHN	chr5	1629558	1629890
SlIDA6	PIPASGPSRKHN	chr11	533816	534061
SlIDA7	GKKPVSGPSKRT	chr6	32587939	32588,208
SlIDA8	PLPPSAPSCRSS	chr9	540110	540304
SlIDA9	PIPPSAPSCRSS	chr9	540110	540220
SlIDA10	PLSPSAPSCRSS	chr3	55943461	55943673
SlIDA11	PVPPSGPSPIGN	chr5	1627379	1627648

**Table 2:** Physicochemical characteristics of *SlIDA* family genes

IDA member	Protein length (aa)	Molecular weight/Da	hydrophobicity	pI
SlIDA1	101	11456.383	−0.084	10.041
SlIDA2	77	8545.945	−0.239	10.951
SlIDA3	79	8967.543	−0.373	10.959
SlIDA4	93	10506.218	−0.413	10.077
SlIDA5	111	12704.135	−0.695	5.698
SlIDA6	82	9585.199	−0.213	12.401
SlIDA7	90	10401.09	−0.496	10.531
SlIDA8	91	10080.821	−0.03	9.2
SlIDA9	89	9822.628	0.142	10.753
SlIDA10	71	7811.218	−0.056	11.213
SlIDA11	97	10998.67	−0.305	9.01

### Analysis of conserved motifs and structural description of the *SlIDA* gene

**Fig. 2:**
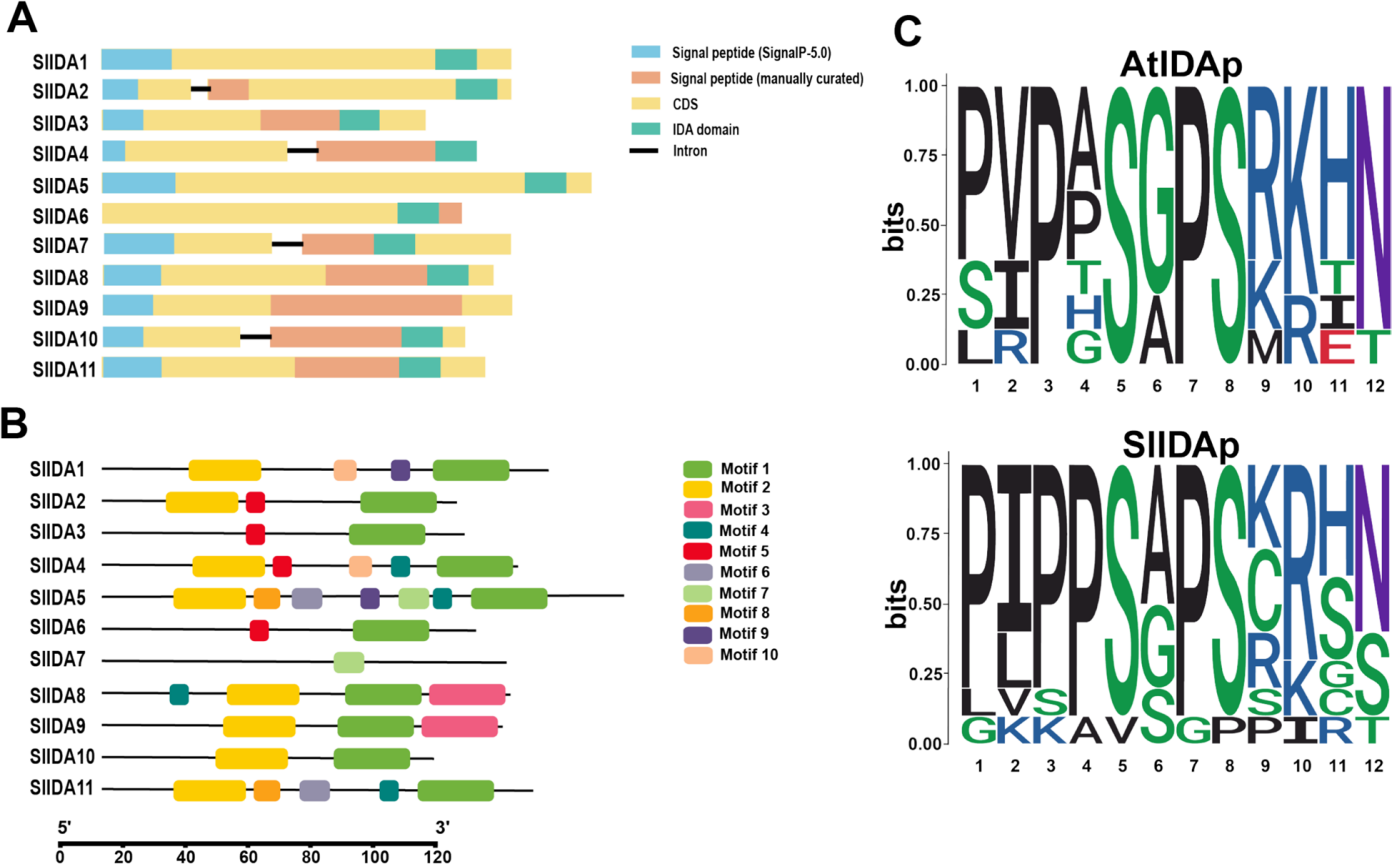
Structural characterization and conserved motif analysis of the *SlIDA* gene. **a** Structures of *SlIDA* genes. **b** Distribution of the conserved motifs in SlIDA proteins. The 1–10 motifs in SlIDA proteins were identified using the MEME program (http://meme-suite.org/) and are displayed in differently colored boxes. **c** Sequence logo of the conserved IDA domain in tomatoes and Arabidopsis using BioLadder (Bioladder. cn). The heights of the bars represent the conserved values of each amino acid at a given position

To further investigate the gene structure of *SlIDA*, we predicted the exon–intron composition of *SlIDA* genes based on sequence homology (Fig. 2a). Overall, the tomato *SlIDA* genes had similar structural types, with only *SlIDA2*, *SlIDA4*, *SlIDA7*, and *SlIDA10* containing introns. In addition, *SlIDA* genes have been hypothesized to exhibit evolutionary diversity based on their structure and length. Furthermore, we identified 10 different motifs (modalities 1–10) in the *SlIDA* genes in the tomato genome using the MEME program (Fig. 2b; Table 3). Visualization and analysis were performed using TBtools software [32]. The results showed that the amino acid lengths of these conserved motifs ranged from 6 to 21 aa and that *SlIDA* genes in the same class had similar conserved motifs. To assess the degree of conservation of *IDA* structural domains between Arabidopsis and tomatoes, we created sequence identifiers (Fig. 2c). We found that the *IDA* structural domains were highly conserved, including proline at positions 1, 3, and 7, serine at position 5, and histidine-asparagine/histidine at positions 11–12.

**Table 3:** Details of conserved motifs in tomato IDA proteins

Motif	Length (aa)	Best possible match
1	21	FFQMLPKGVPIPPSAPSKRHN
2	20	TTTYYSSSKFITLILILSLV
3	21	GTPPSCPMAQFEIVDVVESFT
4	6	NEKKEF
5	6	HHAHGA
6	9	TMNSKEEED
7	9	WSDPKYFDE
8	8	GYASSMRI
9	6	KKEDAY
10	7	EENSRTE

### Phylogenetic analysis

**Fig. 3:**
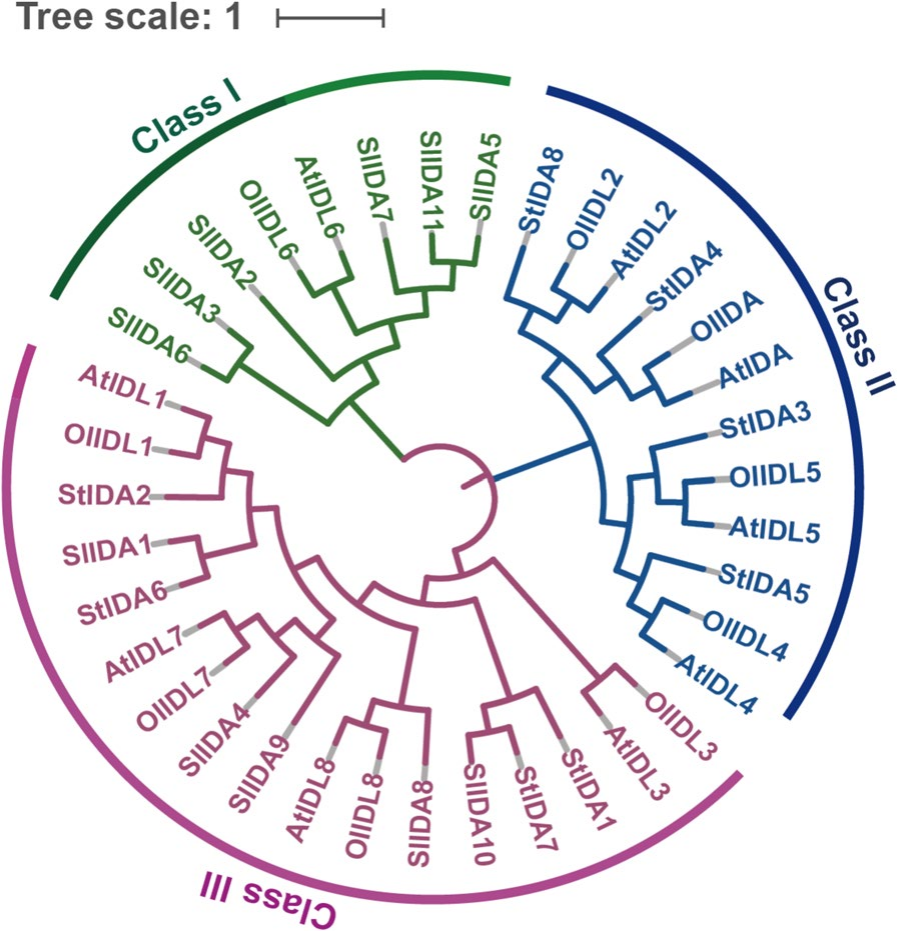
Phylogenetic tree showing the *IDA* gene in *Arabidopsis*, tomato, *tobacco*, and potato. The IDA proteins were classified into three subfamilies and distinguished by different colors

To investigate the evolutionary pattern of *IDA* genes in plants, we performed sequence comparisons using the Tomato Genome Database (https://solgenomics.net/search/locus) and Ensembl Genome (https://plants.ensembl.org/index.html) (Supplementary Table S2) to* o*btain IDA homologous genes of Arabidopsis. Using the *Arabidopsis* IDA gene as a reference, all IDA proteins were classified into three subgroups (I-III). An unrooted phylogenetic tree of IDA protein sequences from four species-tomato, *Arabidopsis*, *Nicotiana tabacum*, and Solanum tuberosum-was constructed using MEGA software (Fig. 3). Two subfamilies were identified in tomato: Subfamily I (SlIDA2, SlIDA3, SlIDA5, SlIDA6, SlIDA7, and SlIDA11) and Subfamily III (SlIDA1, SlIDA4, SlIDA8, SlIDA9, and SlIDA10).

### Expression pattern of the *SlIDA* gene in different tissues of tomato

**Fig. 4:**
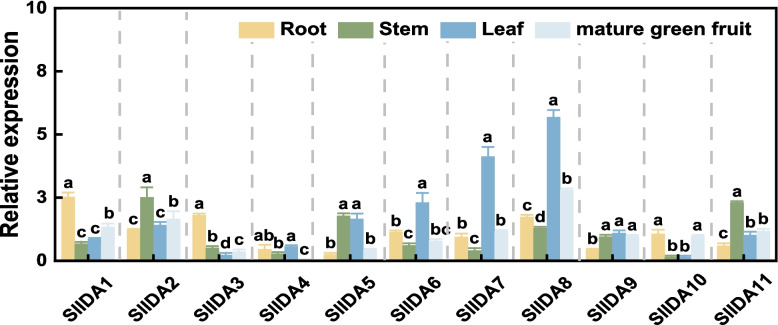
Expression characterization of *SlIDA* gene expression. Expression profiles of *SlIDA* genes in different tissues, including roots, stems, leaves, flowers, and fruits. In the qRT-PCR analysis, ACTIN was used as the internal reference gene, and the relative expression of each gene in tomato flowers was considered as 1. Data are expressed as mean ± SD (*n* = 4). Different letters indicate that the relative gene expression in different tissues was statistically different at the 5% significance level, according to Tukey's pairwise comparison test

To further understand the biological functions of the *SlIDA* genes, we first selected the publicly available RNAseq dataset (http://ted.bti.cornell.edu/cgi-bin/ TFGD/digital/home.cgi) to investigate the expression of the *IDA* gene in different tomato tissue types [33]. Because there were five untagged genes among the 11 *SlIDA* genes, we did not consider them when analyzing the transcriptome data. By analyzing the transcriptome data for the six existing marker genes, we found that the highest expression of *IDA* genes was found in tomato flowers; therefore, we selected flowers as control genes for real-time fluorescence quantification to analyze the relative expression of *IDA* genes. We collected tomato roots, leaves, flowers, stems, and green ripe fruits and analyzed the expression of 11 *SlIDA* genes in these tissues using qRT-PCR. qRT-PCR analysis showed that the 11 *SlIDA* genes had significant tissue-specific expression (Fig. 4). *SlIDA2*, *SlIDA5*, and *SlIDA11* were relatively highly expressed in tomato stems. *SlIDA7* and *SlIDA8* were highly expressed in tomato leaves. The transcriptomic data showed relatively high expression of *SlIDA1*, *SlIDA8*, and *SlIDA9* in flowers, which was consistent with the previously reported function of *IDA* in relation to inflorescence abscission. The expression of *SlIDA1* and *SlIDA3* was relatively high in the roots.

### Screening of *SlIDA* genes in response to salt stress

**Fig. 5:**
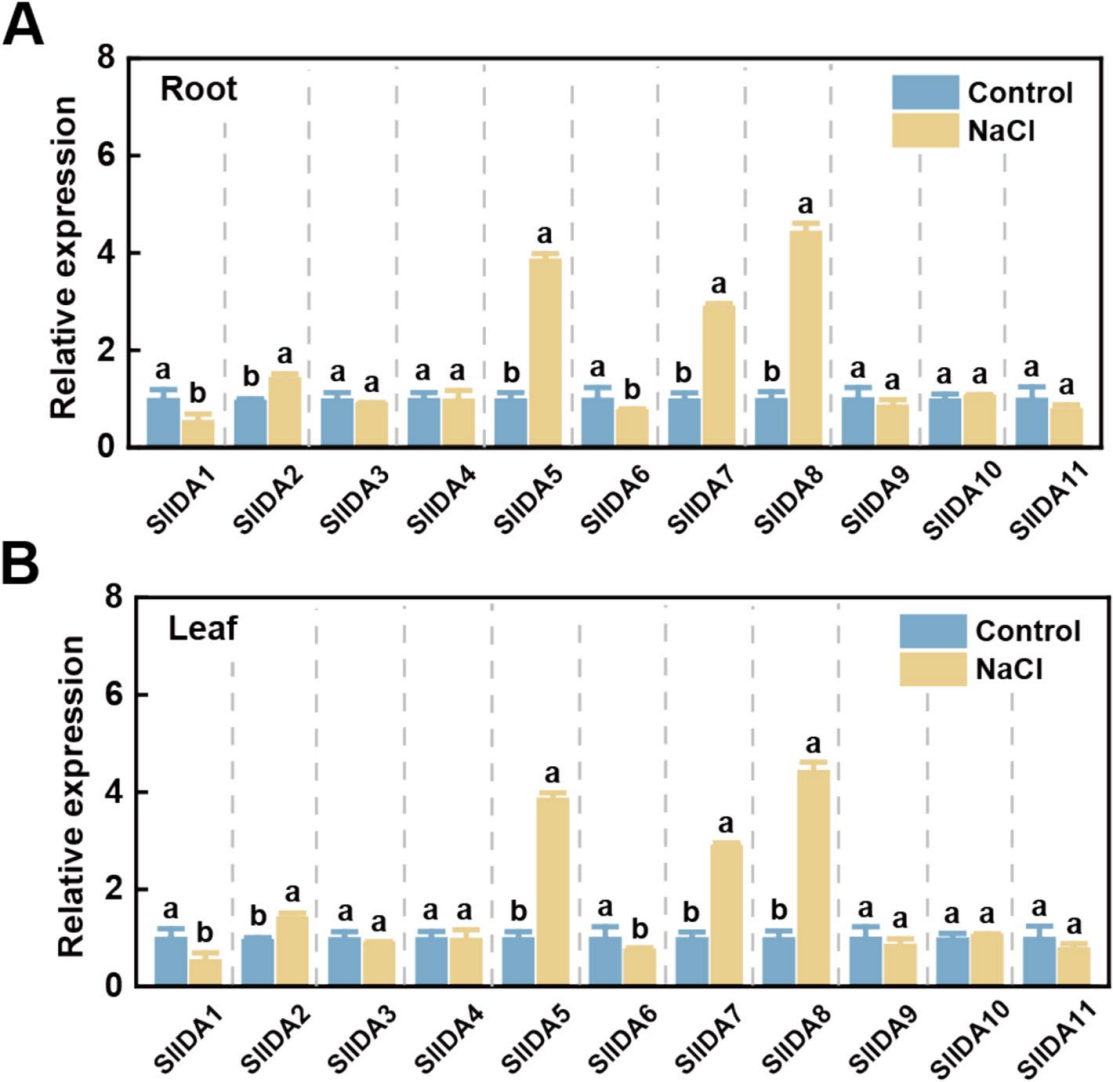
Expression profiles of tomato *IDA* genes under salt stress. **a** Roots; and **b** expression profiles of *SlIDA* in leaves under normal and salt stress conditions. In the qRT-PCR analysis, ACTIN was used as an internal reference gene, and gene expression in tomato roots and leaves under normal conditions was defined as 1. Data are expressed as mean ± SD (*n* = 4). According to the t-test, different letters indicate that the genes were statistically different between the control and treated groups at the 5% significance level

We measured the relative expression of the *IDA* gene in leaves and roots of hydroponically grown tomato seedlings treated with 150 mM NaCl to investigate the response of the *SlIDA* gene to salt stress. qRT-PCR results showed that the relative expression of *SlIDA5*, *SlIDA7*, and *SlIDA8* in tomato roots and leaves increased significantly after salt stress, with *SlIDA8* being the most significantly upregulated (Fig. 5a and b). Therefore, we initially selected these three genes as target genes. VIGS experiments were performed to suppress the expression of *SlIDA5*, *SlIDA7*, and *SlIDA8*, and silenced plants were cultivated in substrate culture. Silenced plants were subjected to root irrigation with 200 mM NaCl, and the phenotype after salt treatment was observed. Compared with other plants, *SlIDA8*-silenced plants exhibited the most sensitive phenotype to salt stress, consistent with the most dwarfism phenotype and the highest electrolyte leakage value (Supplementary Figure S2). These findings suggest that SlIDA8 may be crucial for tomato plants' ability to withstand salt.

### Analysis of the spatial expression pattern of *SlIDA8*

**Fig. 6:**
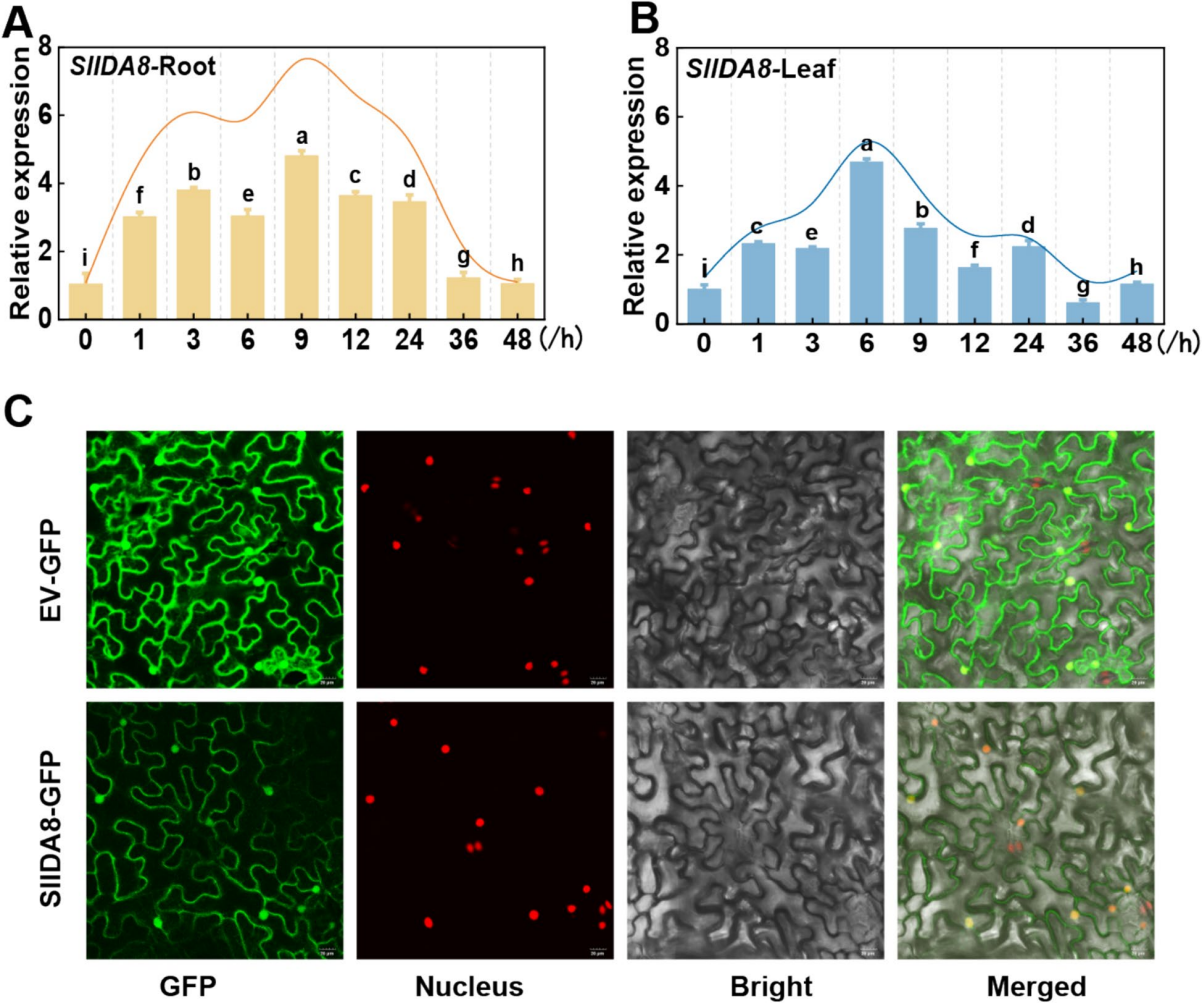
Expression of *SlIDA8* gene in response to salt stress. **a** Leaf. **b** Root. In the qRT-PCR analyses, gene expression in tomato leaves under normal conditions, with ACTIN as the internal reference gene, was defined as 1. **c** SlIDA8-GFP (green fluorescent protein) fusion proteins' subcellular location in tobacco leaf epidermal cells. Data are expressed as mean ± SD (*n* = 4). Different letters indicate that the relative expression of genes at different time points was statistically different at the 5% significance level, according to Tukey’s pairwise comparison test

To further investigate the expression pattern of *SlIDA8*, 4-week-old WT plants grown in hydroponics were treated with 150 mM NaCl, and tomato root and leaf samples were collected at 0, 1, 3, 6, 9, 12, 24, 36, and 48 h after the salt treatment. Total RNA and cDNA synthesized from the root system and leaves were extracted and analyzed using qRT-PCR. The results showed that the expression of *SlIDA8* in the root system and leaves showed a tendency to increase and then decrease with increasing salt treatment time; the maximum expression time point in the root system was 9 h after salt treatment (Fig. 6a), and the maximum expression in the leaves was 6 h after salt treatment (Fig. 6b). To further investigate the subcellular localization of SlIDA8 protein, we constructed a CaMV35S promoter-driven SlIDA8-GFP fusion protein and transiently expressed it in tobacco plants. Confocal microscopy showed that the SlIDA8-GFP fluorescence signal overlapped with that in the cytoplasm. Thus, SlIDA8 proteins were localized to the cell membrane and nucleus (Fig. 6c).

### Inhibition of *SlIDA8* expression reduced salt tolerance in tomato

**Fig. 7:**
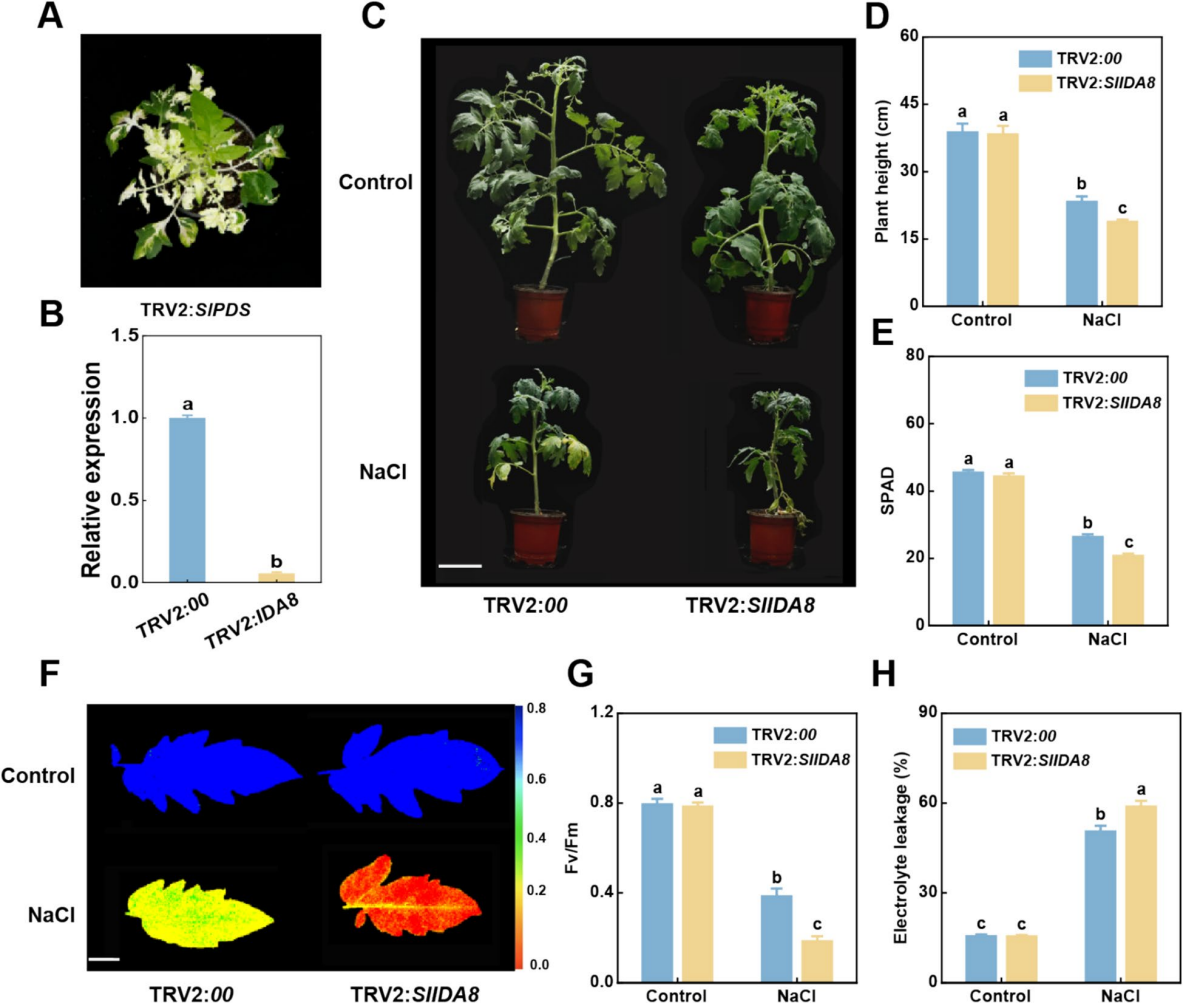
Knockdown of *SlIDA8* reduces salt stress tolerance in tomatoes. **a** Phenotypes of *SlPDS*-silenced tomato plants using VIGS. **b** Expression levels of *SlIDA8* in *SlIDA8*-silenced and control plants. **c** Phenotypes of *SlIDA8*-silenced and control plants treated with NaCl (200 mM). Scale bar, 5 cm. **d** Plant height of control and SlIDA8-silenced tomato leaves. **e** SPAD of *SlIDA8*-silenced and control plants. **f** Fv/Fm of *SlIDA8*-silenced and control plants treated with NaCl (200 mM). Scale bar, 1 cm. **g** Fv/Fm and **h** electrolyte leakage in *SlIDA8*-silenced and control plants. of Data are expressed as mean ± SD (*n* = 4). Different letters indicate statistical differences at the 5% significance level according to Tukey's pairwise comparison test

To further investigate the specific function of *SlIDA8* salt stress in tomato, we used VIGS to inhibit the expression of *SlIDA8*. *SlPDS* plants exhibited a photobleaching phenotype after 15 d (Fig. 7a). According to the results of qRT-PCR, TRV2:*SlIDA8* plants had substantially less *SlIDA8* expression than the control plants (TRV2:*00*), indicating that *SlIDA8* was successfully silenced (Fig. 7b).

When salt stress (200 mM NaCl) was applied after 14 d, TRV2: *SlIDA8* plants clearly showed overall leaf drying and yellowing, indicating that the plants were severely stressed, whereas the control plants showed only yellowing of the lower leaves (Fig. 7c). The heights of the silenced and control plants also differed significantly after salt treatment, with the control plants being 23.55% taller than the silenced plants (Fig. 7d). Moreover, the SPAD values of silenced plant leaves under salt stress were significantly decreased compared with those of the control, with Fv/Fm values only half of those of the control, whereas electrolyte leakage values were increased by 73.33% (Fig. 7e-h). These results suggest that inhibition of *SlIDA8* gene expression reduces salt tolerance in tomatoes.

**Fig. 8:**
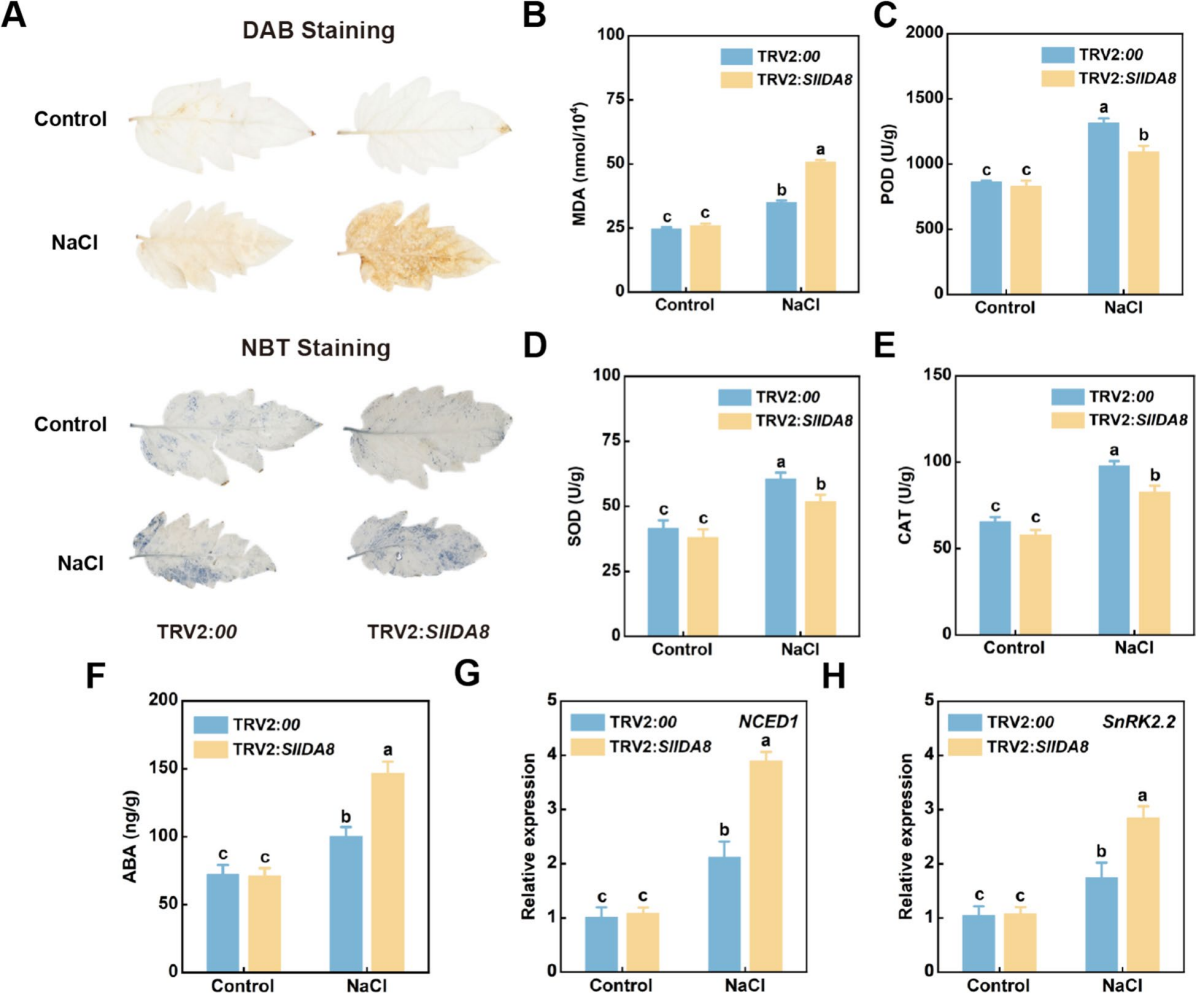
Effects of *SlIDA8* gene knockout on enzyme activities and plant hormone contents in tomato plants under salt stress. **a** Superoxide content in leaves of control and *SlIDA8*-silenced tomatoes detected by DAB and NBT staining. **b** Malondialdehyde content; **c** Peroxidase activity; **d** Superoxide dismutase activity; **e** Catalase activity; **f** ABA content; **g** Relative expression of *NCED* genes; **h** Relative expression of *SnRK2*.2 genes. Data are presented as mean ± standard deviation (*n* = 4). Different letters indicate statistically significant differences between treatment groups and the control group at the 5% significance level according to Tukey's pairwise comparison test

When plants are subjected to salt stress, the balance of ROS, protective enzyme systems, and the balance of osmotic systems in plants is disrupted, and the excessive accumulation of ROS free radicals damages the cell membrane lipid system, which seriously affects the growth and development of plants. Therefore, we assessed the response of *SlIDA8*-silenced plants to salt stress by staining tomato leaves with DAB and NBT and measuring the activities of reactive oxygen scavenging enzymes (POD, SOD, and CAT) and the MDA content in tomato leaves. DAB and NBT staining were performed on the two groups of plants under salt stress, and the results showed that TRV2: *SlIDA8* plants had higher O_2_^−^ and H_2_O_2_ accumulation than control plants, reflecting a greater degree of cell membrane damage in TRV2: *SlIDA8* plants (Fig. 8a) under salt stress at the same salt concentration. In the absence of salt treatment, there was no significant difference in the activity of reactive oxygen scavenging enzymes between the silenced and control plants. Significant changes in reactive oxygen scavenging enzyme activity and MDA content were observed in salt-treated silenced plants compared to those in control plants. Among them, MDA content increased by 48.81%, POD activity decreased by 24.07%, CAT activity decreased by 29.62%, and SOD activity decreased by 26.29% in *SlIDA8*-silenced plants compared to the control group (Fig. 8b-e). Compared to control plants, salt-treated silenced plants exhibited significant changes in ABA content and the relative expression levels of genes involved in ABA synthesis and signal transduction. Specifically, ABA content in SlIDA8-silenced plants increased by 46.13% relative to the control group, *NCED1* relative expression rose by 83.31%, and *SnRK2.2* relative expression increased by 62.6% (Fig. 8f-h). These results suggest that silencing of the *SlIDA8* gene leads to the accumulation of harmful substances in tomato plants, thus reducing their tolerance to salt stress.

## Discussion

Salt stress is a common abiotic stress that seriously affects the growth and development of plants, which in turn affects crop yield and quality, and has a serious impact on agricultural production [2]. There are various ways for plants to cope with salt stress, among which the phytohormone pathway plays a great role in the plant's response to salt stress. Peptides, also considered a new type of phytohormone, have been shown to play an important role as signaling molecules in plant growth, development, and defense [34]. IDA, an inflorescence-deficient abscission peptide, was initially isolated from *Arabidopsis thaliana* and screened for flower abscission-deficient mutants [35]. In an earlier study, researchers identified only five genes encoding peptides similar to IDA in *Arabidopsis*, IDA-LIKE 1–5 (IDL1-5) [16]. With advances in sequencing technology, IDA peptide family members in *Arabidopsis* were identified four more times; in addition, IDA is widely present in dicotyledons and monocotyledons. Moreover, IDA/IDLs have coevolved in monocotyledons and dicotyledons, with a lysine/glycine (K/G) cleavage site at the N-terminal end of the IDA/IDL peptides common in dicotyledons, whereas proline/arginine (P/R) is more prevalent in monocotyledons. Because the last amino acid of most IDA/IDL peptides is asparagine (N), the C-terminus of mature IDA/IDL peptides is highly conserved [36]. Moreover, the seventh proline of PIP was found in all IDA/IDL peptides, revealing the importance of this site. In our study, we used nine IDA peptide genes of *Arabidopsis thaliana* as the target sequences in the Solanum lycopersicum genome (https://solgenomics.ösearch/locus) and Ensembl Genome (https://plants.ensembl.org/index.html) databases for BLAST to identify 11 *IDA* genes in tomatoes, which contained five untagged genes (Fig. 1). After analyzing the IDA-domain sequences of tomato, we found that the tomato IDA polypeptides were highly conserved in the IDA-domain region, and we similarly found that position 7 in the tomato IDA-domain was a proline, suggesting that IDA was highly conserved (Fig. 2c). By visualizing the *IDA* gene structures of tomatoes, we found that all 11 tomato *IDA* genes contained typical IDA-domain structural domains, and the gene structures were relatively simple; only *SlIDA2*, *SlIDA4*, *SlIDA7* and *SlIDA10* had introns (Fig. 2a). Meanwhile, because of the structural characteristics of the IDA polypeptide, the functional fragment was an IDA-domain region consisting of 12 amino acids, and amino acid mutations in the non-IDA-domain region had less impact on the function of the IDA polypeptide. We hypothesized that the function of the IDA polypeptide was also relatively conserved among different tomato varieties. Multiple sequence comparisons revealed that the tomato IDA polypeptide was highly conserved in the IDA domain and poorly conserved in the non-IDA domain (Supplementary Figure S1). Evolutionary analysis showed that the tomato *IDA* gene was classified into two subfamilies (and Ⅲ), and analysis of the IDA-domains of the different subfamilies revealed that only *SlIDA5* in subfamily Ⅰused L (leucine), the *SlIDA7* in subfamily Ⅲ used S (serine). All others start with proline. This finding aligns with evolutionary tree analyses of *rice*, *sorghum*, *maize*, and *Arabidopsis* [12]. Notably, our analysis of cis-regulatory elements in the tomato IDA promoter revealed that the plant hormones abscisic acid (ABA) and methyl jasmonate (MeJA), and salicylic acid (SA) are present in the cis-regulatory elements of the tomato IDA promoter and in abiotic stress response elements (Supplementary Figure S3). These findings strongly suggest that the functions of this gene family may be extensively integrated into plant stress signaling networks, thereby providing crucial clues for elucidating their specific mechanisms in stress responses.

This hypothesis is strongly supported by extensive functional studies indicating that the IDA/IDL peptide family, similar to the known CLE and PSK peptide families, not only mediates plant growth and development processes but also participates in abiotic stress responses [17, 37, 38]. For instance, *CLE* genes play crucial roles in the formation and development of root meristems, vascular tissues, stems, and stomata [39], while *CLE25* and *CLE9* contribute to the dehydration stress response in *Arabidopsis* [39, 40]. PSK induces hydrolytic enzymes in phloem cells and triggers flower abscission under drought stress [41, 42]. Furthermore, analyses of *IDA* gene functions in tobacco and Arabidopsis reveal their extensive involvement in regulating plant growth and development as well as abiotic stress responses [36, 43, 44]. Given the high homology between *tomato* and *Arabidopsis IDA* genes, we hypothesize that *tomato IDA* genes are similarly induced by salt stress. To validate this hypothesis, we first analyzed the expression profiles of this gene family across different tomato tissues. Results revealed distinct tissue-specific expression patterns (Fig. 4): *SlIDA8* exhibited peak expression during green fruit ripening, consistent with its known role in regulating abscission [21], suggesting other family members may harbor undiscovered functions. Subsequently, we analyzed the relative expression levels of this gene family under salt stress (Fig. 5a and b). Three genes exhibited rapid and intense responses. To investigate their functional relevance, we constructed VIGS-silenced plants and subjected them to salt treatment. Results indicated that *SlIDA8*-silenced plants exhibited the most severe salt sensitivity (Supplementary Figure S2). Therefore, based on its most prominent stress-induced expression pattern and most severe silencing phenotype, we identified *SlIDA8* as a key mediator of the tomato salt stress response within this gene family and selected it as the target for subsequent mechanistic studies. Given the central roles of ROS homeostasis and the ABA signaling pathway in salt stress responses, we will investigate whether *SlIDA8* functions by regulating these two critical pathways.

**Fig. 9:**
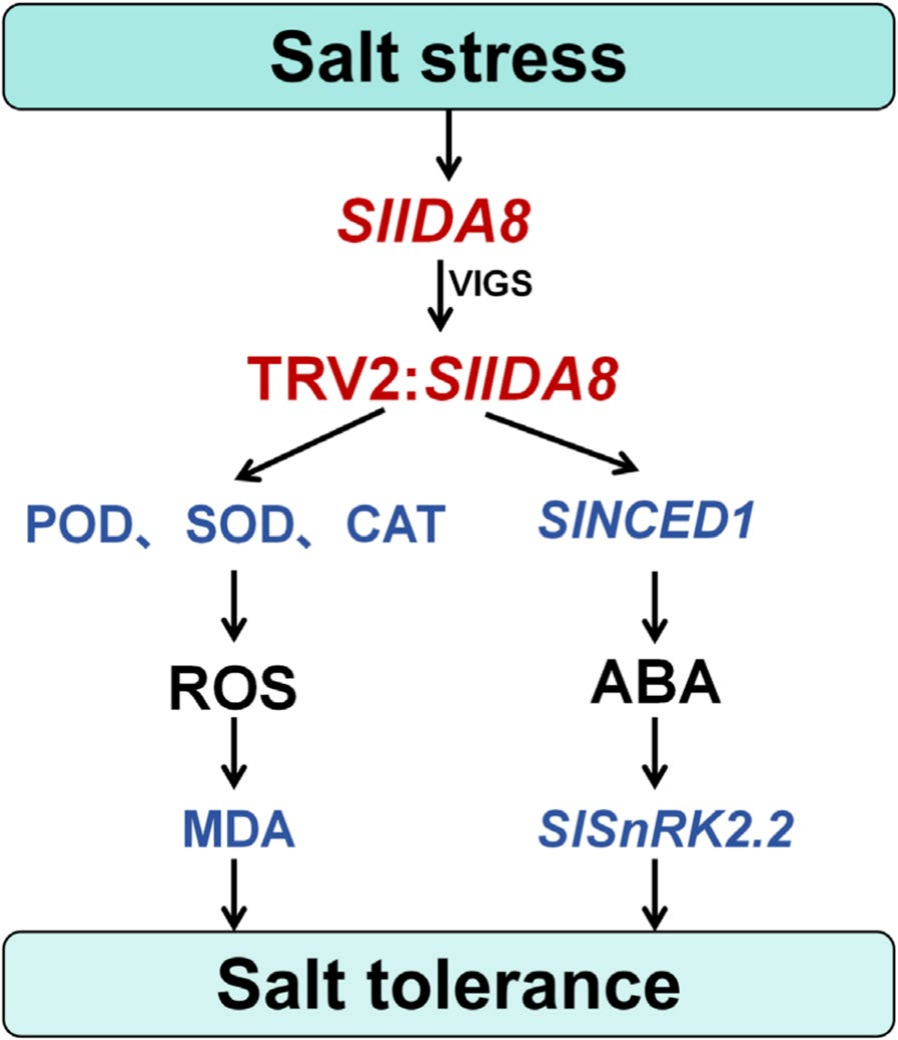
Knockdown of *SlIDA8* reduces tomato salt tolerance. Under salt stress, the antioxidant system in SlIDA8-silenced plants is impaired, enzyme activity decreases, reactive oxygen species accumulate significantly, leading to severe membrane lipid oxidation and damage; ABA-related synthesis and signal transduction gene expression is upregulated, and ABA content accumulates. These changes collectively cause silenced plants to be more sensitive to salt stress, resulting in increased damage

Salt stress triggers an explosion of reactive oxygen species (ROS) within plants, which serve as key signaling molecules but cause oxidative damage when accumulated excessively [28, 45]. Therefore, activating the ROS scavenging system is a core component of plant salt tolerance [28, 46]. Based on this, we investigated whether *SlIDA8* mediates tomato salt stress responses by regulating ROS homeostasis. Additionally, given the close interaction between ROS signaling and the ABA pathway under salt stress [5, 47, 48], we also examined SlIDA8's effects on the ABA pathway. This study revealed that *SlIDA8*-silenced plants exhibited reduced salt tolerance, with salt-treated silenced plants displaying a more dwarf and chlorotic phenotype (Fig. 7c). Furthermore, under salt stress, *SlIDA8*-silenced plants accumulated higher levels of H₂O₂and O₂⁻ compared to controls (Fig. 8a). MDA content was elevated in *SlIDA8*-silenced plants compared to controls (Fig. 8b), indicating more severe membrane lipid peroxidation damage. Correspondingly, the activities of three key antioxidant enzymes (SOD, CAT, and POD) were significantly reduced in *SlIDA8*-silenced plants (TRV2:*00*) (Fig. 8c-e). These results collectively demonstrate that *SlIDA8* deficiency severely impairs tomato ROS scavenging capacity, leading to heightened oxidative damage and reduced salt tolerance. Furthermore, we found *SlIDA8* also modulates the ABA pathway. Under salt stress, endogenous ABA content increased, and expression of key ABA biosynthesis and signaling genes *NCED1* and *SnRK2*.2 was significantly upregulated (Fig. 8f-h). Integrating these findings, we propose a working model: under salt stress, *SlIDA8* maintains ROS homeostasis by positively regulating antioxidant enzymes to mitigate oxidative damage. Concurrently, it likely suppresses excessive ABA pathway activation through negative feedback regulation, enabling more precise stress adaptation at the signaling level (Fig. 9). These synergistic actions collectively determine tomato salt stress tolerance. This study provides preliminary insights into a novel mechanism by which *SlIDA8* mediates salt stress responses through the integration of ROS and ABA signaling networks.

In *Arabidopsis*, IDA/IDL peptides are recognized by a receptor complex comprising the receptor HAE/HSL1/HSL2 and co-receptor SERKs, triggering a downstream mitogen-activated protein kinase (MAPK) cascade involving three layers of protein kinases [49, 50]. Although this interaction has not been directly validated in tomato, given the high sequence and functional conservation between tomato and *Arabidopsis* homologs, we reasonably speculate that SlIDA8 may perceive and transmit salt stress signals through a similar mode of action, by binding to a homologous tomato LRR-RLK receptor-thereby activating the aforementioned ROS and ABA responses. This hypothesis provides a clear research direction for elucidating the complete mechanism of action of this gene, but requires subsequent protein interaction and genetic experiments for validation.

In summary, *SlIDA8* gene expression is upregulated when tomatoes undergo salt stress. In *SlIDA8*-silencing plants, the antioxidant enzyme system becomes dysfunctional under salt stress, manifested by decreased SOD, POD, and CAT enzyme activities. This leads to weakened reactive oxygen species scavenging capacity, elevated MDA content, accelerated membrane lipid peroxidation, and damage to the cell membrane system. Concurrently, ABA synthesis increases, and the expression of ABA-related synthesis and signal transduction genes is up-regulated, suggesting that the *SlIDA8* may negatively regulate ABA accumulation or signal transduction. Collectively, these changes render *SlIDA8*-silenced plants more sensitive to salt stress, resulting in heightened damage (Fig. 9).

Furthermore, while VIGS technology serves as a powerful tool for rapid gene silencing, its transient nature and potential variability in silencing efficiency mean that the resulting phenotypic outcomes should be regarded as strong indicative evidence rather than definitive conclusions. Therefore, our future experiments will focus on systematically constructing and validating stable genetic materials for SlIDA8, particularly by generating knockout mutants through gene editing techniques and establishing stable transgenic lines with high-efficiency overexpression vectors. The construction of these genetic resources will provide essential tools for elucidating the biological function of SlIDA8 in response to salt stress. This work will not only determine whether this gene directly regulates salt tolerance but also lay a solid foundation for further investigating its dynamic role in plants' long-term adaptation to salt stress.

## Conclusions

In summary, IDA polypeptides are widely distributed in eukaryotes, and IDA proteins in *Arabidopsis thaliana* and tomato have high homology and similar functions. Analysis of the relative expression of *IDA* family genes in tomato under salt stress confirmed that the *SlIDA8* gene has a regulatory role in salt stress, and the VIGS experiment further confirmed that *SlIDA8* regulates the response of tomato to salt stress through the ROS pathway. The above results preliminarily revealed the function of *SlIDA8* in regulating salt tolerance and provided a basis for further elucidation of the function of *IDA* family genes. This research holds significant importance for elucidating the functional mechanisms of the tomato IDA peptide family and provides new insights into tomato responses to salt stress.

## Supplementary Information

Supplementary Material 1. Figure S1. Multiple sequence alignment of SlIDA proteins in tomatoes. Figure S2. Salt sensitivity of the VIGS plants. Treatment of *SlIDA5*, *SlIDA7*, and *SlIDA8* silenced plants with 200 mM NaCl showed that *SlIDA8*-silenced plants were more sensitive to salt stress. Figure S3. Cis-regulatory elements in the promoter regions of *SlIDA *genes. Figure S4. Expression profiles of 11* SlIDA* genes during salt stress. The heatmap displays the relative expression levels of all 11 *SlIDA* genes in hydroponic tomato leaves at 0, 3, 6, and 12 hours after treatment with 150 mM NaCl. The blue-to-red color scale indicates low to high expression levels.

Supplementary Material 2. Table S1. Primers used in this study. Table S2. Gene sequence.

## Data Availability

All data supporting the findings of this study are available within the paper and its Supplementary Information.

## Abbreviations

IDA: Inflorescence Deficient In Abscission
ABA: Abscisic acid
JA: Jasmonic acid
SA: Salicylic acid
MeJA: Methyl Jasmonate
ETH: Ethylene
HAE: HAESA
HSL2: HAESA-L1KE2
VIGS: Virus-induced gene silencing
MDA: Malondialdehyde
POD: Peroxidase catalase
CAT: Catalase
L: Leucine
S: Serine
P: Proline
