# Supplementary material for: Identification of the IDA peptide family in tomato and function of SlIDA8 in salt stress
Source: BMC Plant Biol. 2026 Jan 20;26:300. doi: 10.1186/s12870-026-08179-5 (PMC12903503; doi:10.1186/s12870-026-08179-5)
Supplement: Supplementary file 1 — Supplementary Material 1. Figure S1. Multiple sequence alignment of SlIDA proteins in tomatoes. Figure S2. Salt sensitivity of the VIGS plants. Treatment of SlIDA5, SlIDA7, and SlIDA8 silenced plants with 200 mM NaCl showed that SlIDA8-silenced plants were more sensitive to salt stress. Figure S3. Cis-regulatory elements in the promoter regions of SlIDA genes. Figure S4. Expression profiles of 11 SlIDA genes during salt stress. The heatmap displays the relative expression levels of all 11 SlIDA genes in hydroponic tomato leaves at 0, 3, 6, and 12 hours after treatment with 150 mM NaCl. The blue-to-red color scale indicates low to high expression levels. [file 12870_2026_8179_MOESM1_ESM.pptx]

## Slide 1
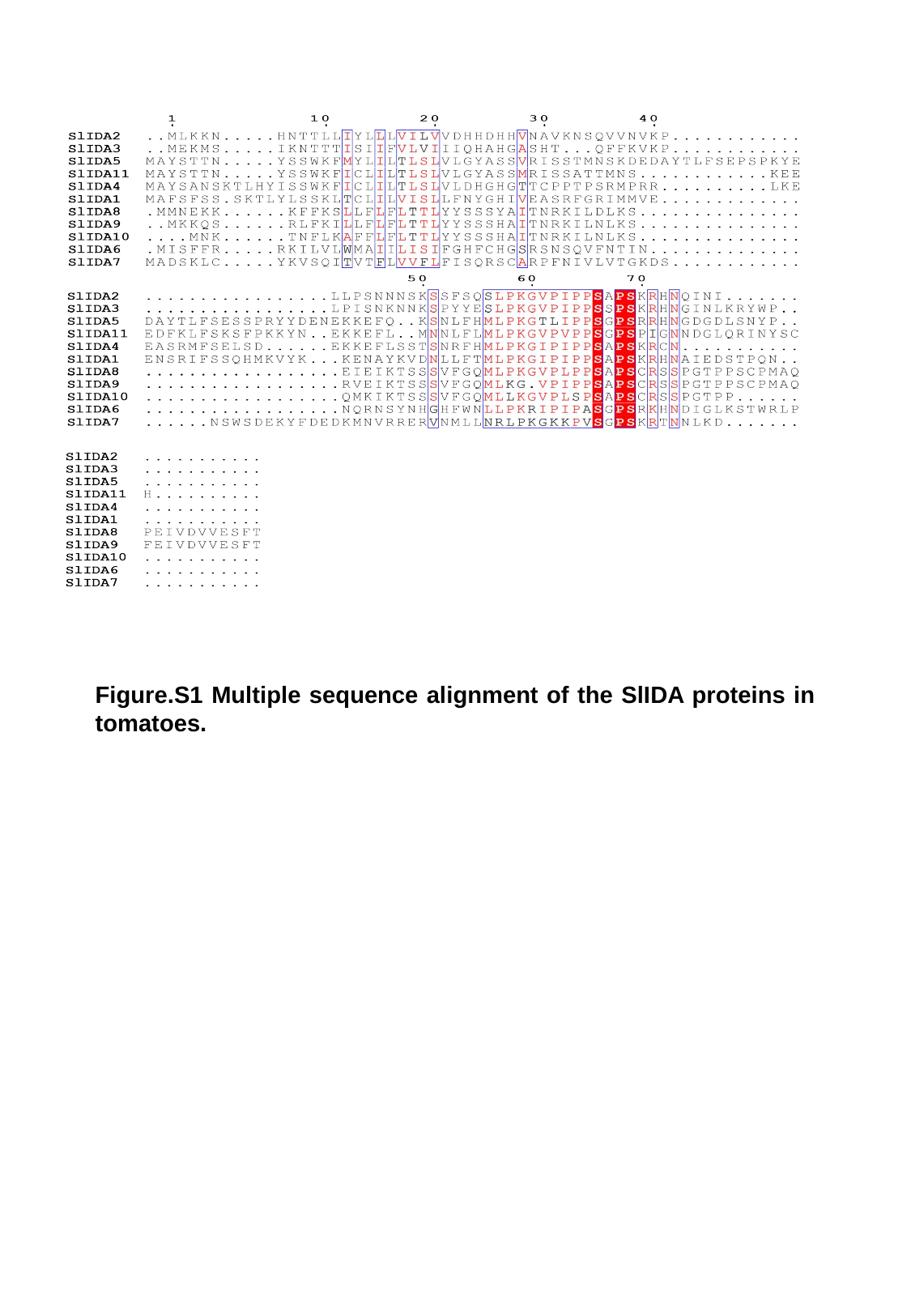

Figure.S1 Multiple sequence alignment of the SlIDA proteins in tomatoes.

## Slide 2
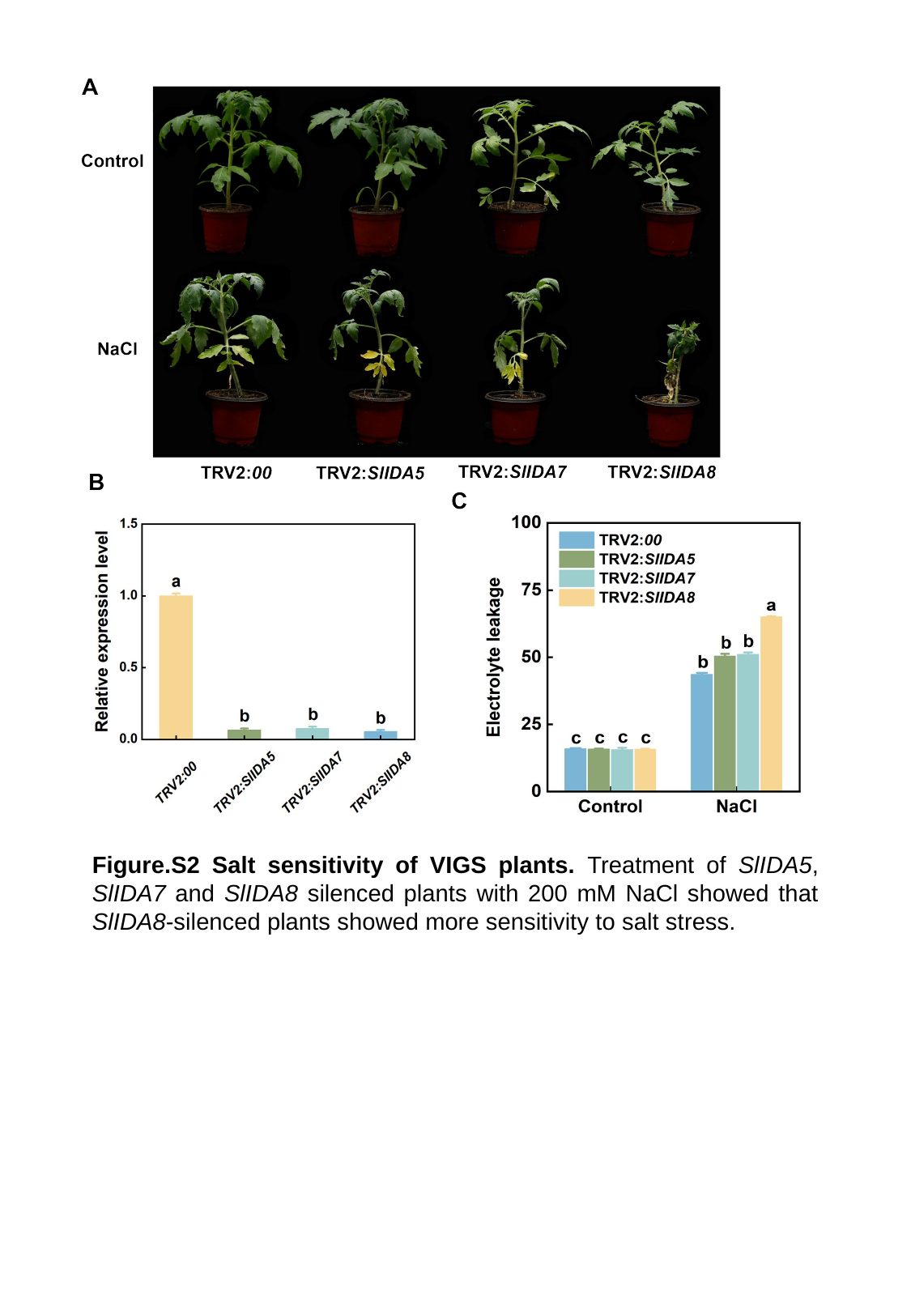

Figure.S2 Salt sensitivity of VIGS plants. Treatment of SlIDA5, SlIDA7 and SlIDA8 silenced plants with 200 mM NaCl showed that SlIDA8-silenced plants showed more sensitivity to salt stress.

## Slide 3
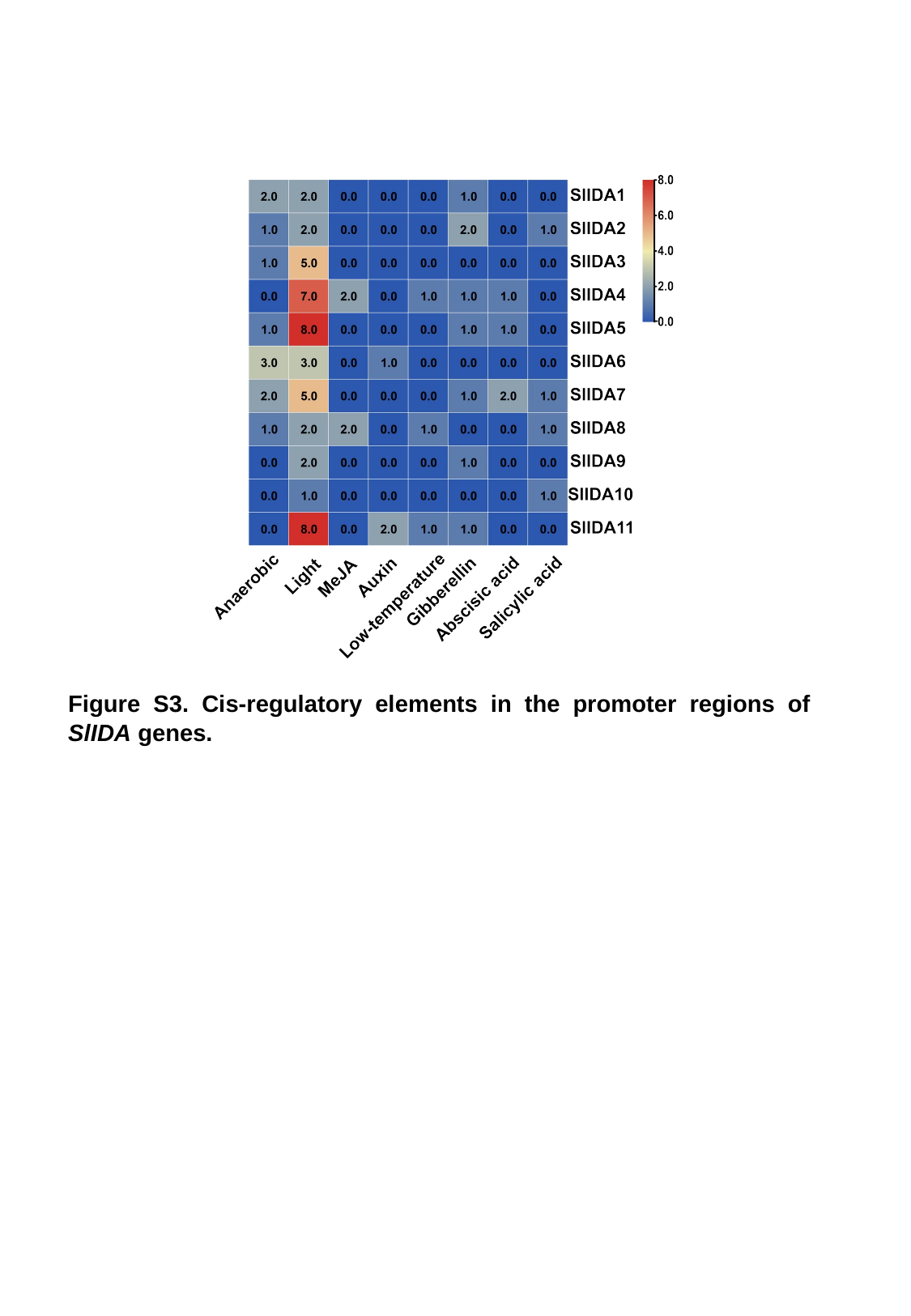

Figure S3. Cis-regulatory elements in the promoter regions of SlIDA genes.

## Slide 4
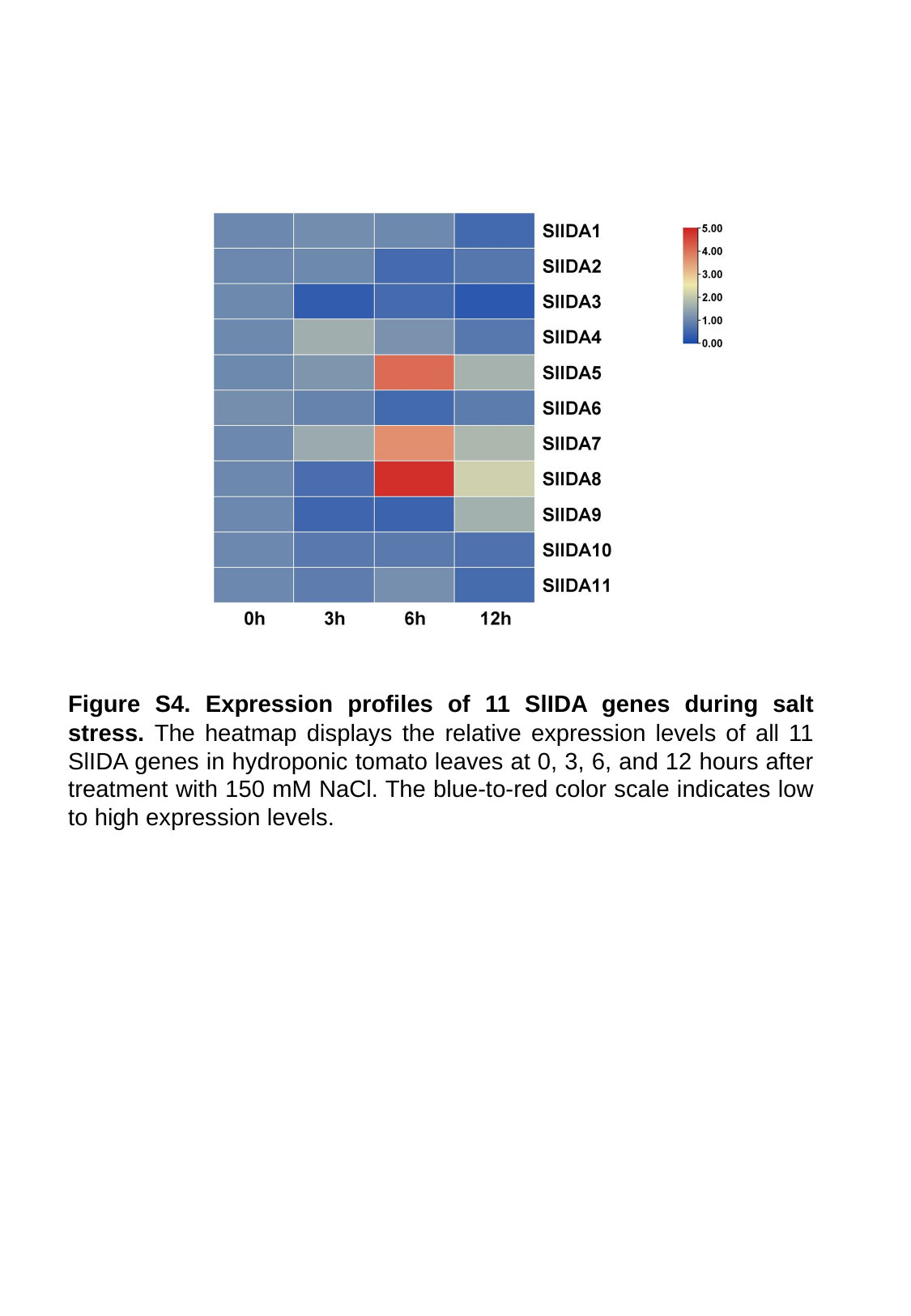

Figure S4. Expression profiles of 11 SlIDA genes during salt stress. The heatmap displays the relative expression levels of all 11 SlIDA genes in hydroponic tomato leaves at 0, 3, 6, and 12 hours after treatment with 150 mM NaCl. The blue-to-red color scale indicates low to high expression levels.
